# Methodological Considerations for Studies in Sport and Exercise Science with Women as Participants: A Working Guide for Standards of Practice for Research on Women

**DOI:** 10.1007/s40279-021-01435-8

**Published:** 2021-03-16

**Authors:** Kirsty J. Elliott-Sale, Clare L. Minahan, Xanne A. K. Janse de Jonge, Kathryn E. Ackerman, Sarianna Sipilä, Naama W. Constantini, Constance M. Lebrun, Anthony C. Hackney

**Affiliations:** 1 Sport Health and Performance Enhancement (SHAPE) Research Centre, Department of Sport Science, Nottingham Trent University, Nottingham, UK; 2 Griffith Sports Science, Griffith University, Gold Coast, QLD Australia; 3 School of Environmental and Life Sciences, The University of Newcastle, Ourimbah, NSW Australia; 4 Division of Sports Medicine, Boston Children’s Hospital; Neuroendocrine Unit, Massachusetts General Hospital, Harvard Medical School, Boston, USA; 5 Gerontology Research Centre and Faculty of Sport and Health Sciences, University of Jyväskylä, Jyväskylä, Finland; 6 Heidi Rothberg Sport Medicine Centre, Shaare Zedek Medical Center, Hebrew University of Jerusalem, Jerusalem, Israel; 7 Glen Sather Sports Medicine Clinic and Department of Family Medicine, Faculty of Medicine and Dentistry, University of Alberta, Alberta, Canada; 8 Department of Exercise and Sport Science; Department of Nutrition, University of North Carolina, Chapel Hill, NC USA

## Abstract

Until recently, there has been less demand for and interest in female-specific sport and exercise science data. As a result, the vast majority of high-quality sport and exercise science data have been derived from studies with men as participants, which reduces the application of these data due to the known physiological differences between the sexes, specifically with regard to reproductive endocrinology. Furthermore, a shortage of specialist knowledge on female physiology in the sport science community, coupled with a reluctance to effectively adapt experimental designs to incorporate female-specific considerations, such as the menstrual cycle, hormonal contraceptive use, pregnancy and the menopause, has slowed the pursuit of knowledge in this field of research. In addition, a lack of agreement on the terminology and methodological approaches (i.e., gold-standard techniques) used within this research area has further hindered the ability of researchers to adequately develop evidenced-based guidelines for female exercisers. The purpose of this paper was to highlight the specific considerations needed when employing women (i.e., from athletes to non-athletes) as participants in sport and exercise science-based research. These considerations relate to *participant selection criteria* and *adaptations for experimental design* and address the diversity and complexities associated with female reproductive endocrinology across the lifespan. This statement intends to promote an increase in the inclusion of women as participants in studies related to sport and exercise science and an enhanced execution of these studies resulting in more high-quality female-specific data.

## Key Points

**Table:** 

Not all ‘women’ are the same. Women have a variety of reproductive hormonal profiles that change across the lifespan from puberty to the menopause.
The endogenous hormonal profile of women is frequently influenced by exogenous sources, such as hormonal contraceptives (HC) and hormone replacement therapy (HRT).
Depending on the research question, women should be recruited on pre-defined, standardised criteria, which, in most cases, should be retrospectively confirmed (i.e., homogenous a priori inclusion and a posteriori exclusion criteria).
Depending on the research question, the experimental design needs to be adapted in line with the hormonal milieu (e.g., consideration of menstrual cycle phase, type of HC used, stage of menopause).

## Introduction

### Statement of the Problem

**Fig. 1:**
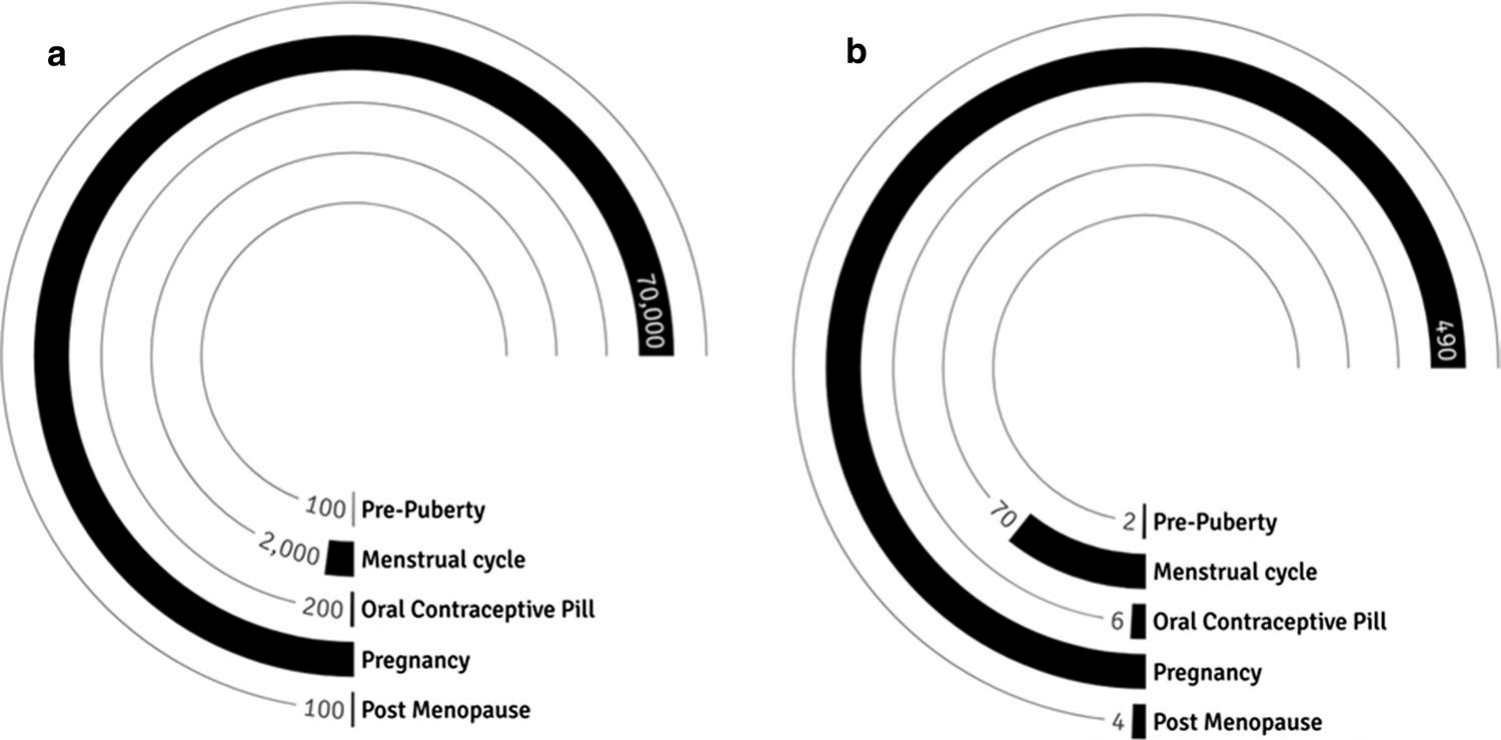
Indicative oestrogen and progesterone profiles across the lifespan from childhood to senescence: **a** oestrogen (pmol∙L^−1^). With regards to oestrogen, the menstrual cycle has 20 times more oestrogen than pre-puberty and pregnancy 35 times more oestrogen than the menstrual cycle; **b** progesterone (nmol∙L^−1^). The menstrual cycle has 35 times more progesterone than pre-puberty and pregnancy almost 7 times more progesterone than during the menstrual cycle. Oral contraceptives users and post-menopausal women have similar levels of endogenous oestrogen and progesterone. Hypothalamic-pituitary forms of amenorrhea (not depicted here) do not show any evidence of oestrogen production based on urinary measurements [1]. In women, “oestrogen” includes the oestrone, oestradiol, oestriol hormones

Across the lifespan, circulating concentrations of oestrogen and progesterone change from puberty, through adulthood, until menopause, when a dramatic decline in the secretion of ovarian steroids occurs and cyclic oestrogen production is replaced by a low constant production by the ovaries. Furthermore, between puberty and menopause, circulating concentrations of oestrogen fluctuate fivefold and progesterone greater than 50-fold over a 21- to 35-day cycle (i.e., menstrual cycle). Indeed, these cyclical acute fluctuations in ovarian hormones across the menstrual cycle may be altered by external factors (e.g., hormonal contraceptives [HC], circadian variation, diet, exercise), clinical conditions (e.g., functional hypothalamic amenorrhea, polycystic ovary syndrome) and pregnancy (Fig. 1; [1]).

In addition to reproductive function, ovarian steroids have been shown to target a number of tissues (e.g., epithelial, connective, muscle and nervous; [2–4]) and influence an array of biological processes (e.g., metabolism, ventilation, immunity, cognition, gastrointestinal, cardiovascular, autonomic regulation, and genitourinary function; [5–13]). As such, different concentrations of circulating ovarian steroids throughout the lifespan and across the menstrual cycle (with and without dysfunction and/or manipulation) might significantly influence the mechanisms that control and regulate cell function and integrated physiologic adaptation in women.

Given the potential influence of ovarian hormones on multiple biological systems, it is reasonable to assume that the heterogeneic findings observed among studies conducted with women as participants, particularly those examining physiological responses to exercise and adaptations to training, may be attributable to the numerous hormonal profiles a woman may experience during her lifetime. As such, it is important that scientists consider the potential effects of ovarian hormones throughout the lifespan on exercise responses and sports performance and apply suitable methodology to their studies in order to control these effects and reduce the heterogeneic results. Nonetheless, for decades scientists have avoided conducting sport and exercise research with women as participants due, in part, to the complex methodological considerations required and/or the difficulty in interpreting the heterogeneic results often observed within and among studies. Consequently, research findings from sport and exercise performance and training studies with men only as participants are applied to women, which can result in a missed opportunity for women to reach their athletic and performance potential. In order to guide scientists and practitioners through the complexities of: *i*. identifying the maturation status and menstrual-cycle status of women participants, and *ii*. designing studies that provide insight into the difference in the physiological responses to exercise among the various hormonal profiles of women, guidance on the definition(s) of sampled populations is required and a working guide for standards of practice for research in this field is warranted.

### Current State of the Art

Research into the effects of the menstrual cycle on the exercise response has being ongoing since at least 1876, when Professor Mary Jacobi won the Harvard University Boylston Prize Essay for her observations on menstruation and physical work and rest [14]. Despite a recent growing interest in research in sport and exercise science with women as participants, the overall research quantity in this area is still very small compared to research with men as participants [15]. Therefore, the effects of oestrogen and progesterone on women’s responses to exercise and adaptations to training are still not fully understood. As a result, there are no sport and exercise-related guidelines for training and nutrition which are customised for sportswomen (i.e., athletes and exercisers) and underpinned by sufficient, high-quality scientific evidence. Numerous reviews [e.g.*,* 16–20], books [e.g., 21–23] and meta-analyses [e.g., 24,25] have summarised and examined data from this field spanning almost 50 years, but all have concluded that more, better quality studies are needed before a consensus on the effects of ovarian steroids on training and performance can be reached.

It is clear that more research focusing on areas unique to female physiology is required to inform best practice guidelines for training and nutrition [24, 25]. There are some studies, however, that have demonstrated clear and recurring findings of ovarian-steroid interference with biological processes that may affect athletic performance (i.e., responses to exercise and adaptation to, and recovery from, training), which highlight the importance of considering ovarian steroid profiles in sport and exercise science research. In particular, the manipulation of endogenous ovarian steroid levels with synthetic hormones (i.e., HC) has been repeatedly associated with increased markers of muscle damage in women after unaccustomed eccentric exercise [8, 26–30]. Therefore, knowledge of the use of HC by athletes or research participants is imperative in understanding the recovery process after an acute bout of exercise or adaptation to training. There is a strong body of evidence indicating altered thermoregulation in women taking HC during exercise including altered skin blood flow and cutaneous vasodilation, as well as a delayed onset of sweating and a higher internal temperature threshold [31–35]. As such, ovarian hormone interference via the administration of HC may have implications for exercise in the heat. Oestrogen is often associated with substrate oxidation and utilisation during prolonged exercise. The notion that elevated plasma oestrogen concentrations increase the reliance on fat as an energy source during endurance exercise is a consistent observation [6, 36–38]. Despite these few convincing findings, whether disturbances to these physiological mechanisms result in a decrement to performance in strength, speed, and/or endurance events remains to be elucidated. It should be also noted that there is always at least one study that opposes the findings of the majority and as newer formulations of synthetic hormones are developed, there will be further ambiguity in the findings. Therefore, the importance of considering a woman’s current ovarian hormonal profile in sport and exercise science research is paramount.

### The Gap in the Field

Rather than viewing the dynamic profiles of ovarian steroids in women as a reason to exclude them from sport and exercise science research, future studies need to explore the specific effects of oestrogen and progesterone and related hormonal ageing on physiological function and ergo athletic performance across the lifespan in women. Female-specific research is needed as the number of women participating in sport and exercise is increasing in many countries, and parity in the Olympics and Paralympics is approaching (i.e., equal number of male and female competitors and medals). For example, in 1900 only 2.2% of the competitors in the Olympics were female; in 1952 the proportion was 10.5%, in 2000 it was 38.2% and in 2021 it is predicted to be 48.8%. If male-based research continues to be applied to females, there is a risk that the true potential of women will not be achieved as the potential influence of ovarian hormones remains unclear; herein exist the gap in the field and the fundamental statement of the problem within contemporary sport and exercise science. At the elite level, understanding the effects of oestrogen and progesterone on body systems and performance may produce the marginal gain needed for success. In addition, there is a need to understand the effects of training on hypothalamic, pituitary and ovarian hormones and related outcomes, such as body composition and bone [39]. At the recreational level, understanding the impact of changes in ovarian hormone concentration, experienced during the menstrual cycle or as a result of menstrual irregularities, hormonal contraceptive use, pregnancy or the menopause, on the ability and willingness to undertake exercise and physical activity may help reduce the disproportionate decline in sports participation seen at puberty in girls [40, 41]. This decrease in physical activity often extends into adulthood, and is compounded during pregnancy and the postpartum period [22], and as such has implications for health and well-being [42] for women of all ages. Among middle-aged women, menopausal hormonal changes are associated with deterioration of the body systems [43], performance and functioning [44], but further understanding is needed of the magnitude and timing of these changes as well as the role of physical activity and exercise to overcome or attenuate these changes.

In order to facilitate a new generation of quality research in sport and exercise science with women as participants that addresses the current gap in the field, direction is needed on the best and most accurate types of research design to employ in this field. In addition, guidance on the definition(s) of sampled populations is required in order to improve the uniformity of studies involving women. As such, this article is intended to serve as a working guide for standards of practice for research on women.

Synchronous with changes in performance, ovarian steroids exert many clinical effects, which have also received less attention in sport and exercise medicine-based research. Examples include hyponatremia, which is more prevalent in females than males [45] and heat stroke, which is influenced by ovarian hormonal concentrations [46]. Although this paper is focused on studies related to sport and exercise science, some of the approaches described herein may be also relevant for female-based sport and exercise medicine-based research.

### Background to the Statement

In the early 2000s, articles started to provide critical commentary on the methodological approach used in studies investigating the effects of oestrogen and progesterone on different aspects of exercise performance [18, 47]. This began to raise an awareness of some of the technical flaws and issues within this field. Cable and Elliott [47] focussed on the methodological approach used in studies investigating the effects of ovarian steroids on muscle strength in women. They identified inconsistencies in terminology and research design, namely differences in the definition of reproductive status (e.g.*,* inconsistencies in menstrual cycle phase definition), often leading to the grouping of non-homogeneous participants; and quantification of reproductive status, by using inaccurate and subjective methods. In addition, they highlighted that consideration needed to be given to characteristics of the hormonal milieu; such as, if the changes in ovarian steroids were acute (e.g., menstrual cycle) or chronic (e.g., menopause); within normal (e.g., menstrual cycle) or supra-physiological (e.g., pregnancy) ranges; caused by endogenous fluctuations (e.g., menstrual cycle) or exogenous (e.g., HC or hormone replacement therapy [HRT]) supplementation. The review by Janse de Jonge [18] focussed on methodological considerations in menstrual cycle research, including the verification of menstrual cycle phase and the timing of testing throughout the cycle. More recently, Janse de Jonge et al. [48] consolidated the approaches and issues observed specifically in menstrual-cycle studies and proposed methodological guidelines for undertaking menstrual cycle-based research in sport and exercise science. Furthermore, broad methodological considerations for studies in exercise endocrinology have been provided lately [49], including the critical consideration of sex and the menstrual cycle. The current statement builds upon these reviews by providing guidance for studies including women from across the lifespan.

### Approach to the Statement

The considerations outlined herein are based on previous research and expert opinion and are delivered alongside the rationale and pros and cons for each method. Where appropriate the primary source has been provided; however, some items have been specifically developed for this statement. For ease of reading we are using oestrogen throughout the paper, but recognise this encompasses oestradiol, oestrone and oestriol. For more detailed information on female endocrinology see Hackney [21].

### Purpose of the Statement

The aim of this statement was to consolidate the opinions, lessons and evidence from experienced researchers in the area of women’s physiology, with a view to providing counsel for those beginning to pursue research in this very important area. Moreover, the considerations described herein are intended to help dispel the myths and poor practices commonly associated with this subject area, thus improving the quality of future research and allowing more meaningful comparisons to be made between studies that have employed consistent terminology and research designs. Overall, this statement aims to contribute to advancing the scientific community’s knowledge and understanding of women’s physiology in the context of sport and exercise science.

## Considerations

### Participants

Much of the heterogeneity in this area of research is derived from the lack of uniformity when describing the populations used in studies with women as participants. While chronological age is often controlled and reported, maturation status and menstrual-cycle status (*i.e*., length, irregularities/dysfunction, manipulation) are more often not. Moreover, if menstrual-cycle status is even considered by researchers, the hormonal profile of the participants is often implied rather than confirmed [25] which may exacerbate the variability between studies.

In order to overcome these issues, consistency is needed in the following: (i) the terminology used to describe participants; and (ii) the inclusion/exclusion criteria, specifically the hormonal parameters, used to define eligible participants. This uniformity will allow meaningful comparison within and among studies (i.e., comparing like with like). Table 1 proposes a framework of terms and conditions, based on previous research and our collective experience and expertise in the field that could help to ensure consistency in women’s sport and exercise-based studies. Across the lifespan, the ovarian steroids start changing at puberty. Therefore, the stage of puberty needs to be classified consistently in research with younger populations. During the reproductive years there are five main factors that will influence the fluctuations in oestrogen and progesterone: i) fluctuations across the eumenorrheic menstrual cycle; ii) disruptions to the eumenorrheic menstrual cycle caused by illness or disease (e.g., polycystic ovary syndrome, endometriosis or other hypothalamic pituitary axis disruptions), low energy availability, the first two years after menses, and the time nearing menopause; iii) manipulation of the eumenorrheic menstrual cycle with exogenous hormones, both synthetic and natural (e.g., HC and HRT); iv) pregnancy (increasing ovarian steroids profile); and v) menopause (decreasing ovarian steroids profile). Researchers should strive to adopt all recommendations that are appropriate, thus facilitating good practice in this area.

**Table 1:** List of the considerations related to participant characteristics and selection criteria

Consideration	Rationale (intended to…)	Pros (could…)	Cons (could…)
Define *puberty* as the onset of menarche [50]	Increase accuracy and validity of the population definition	Reduce between study variability in describing the population studied	Increase timescale of the study in order to recruit participants who fit the criteria
Define *peri-puberty* as the time around puberty			Need to be aware of other physical and endocrine indicators of puberty outside of menarche (e.g.*,* maturation of the genital organs, development of secondary sex characteristics; Tanner stages; oestrogen and progesterone concentrations)
Define *adrenarche* as activation of adrenal androgen production; usually occurs before gonadarche [51]	Increase homogeneity of hormonal profiles Increase consistency of terminology	Reduce between participant variability in hormone status Reduce between study variability in describing the population studied	Reduce availability of eligible participants Increase timescale of the study if the condition needs to be confirmed prior to the commencement of data collection Increase number of participants who need to be excluded (during or retrospectively) from the study if the condition was not confirmed prior to commencement of the study
Define *gonadarche* as activation of reproductive glands leading to menarche [51]			
Define *primary amenorrhea* as failure to reach menarche by age 15 years when development of secondary sexual characteristics is evident, or by age 14 years when no secondary sexual characteristics are present [52, 53]			
Define *eumenorrhea* as menstrual cycle lengths ≥ 21 days and ≤ 35 days resulting in 9 or more consecutive periods per year, plus evidence of LH surge, plus correct hormonal profile, plus no HC use 3 months prior to recruitment			
Define *anovulatory* as those who experience menstruation but do not ovulate (ovulation cannot be detected by urinary LH surge or confirmed by hormone concentrations via blood sample analysis) [49, 55]			
Define *luteal phase deficiency* as cycles with less than 16 nmol·L^−1^ of progesterone, when a single luteal phase progesterone measurement is taken [48]			
Define *oligomenorrhea* as those with cycle length > 35 days [54]			
Define *secondary amenorrhea* as the absence of ≥ 3 consecutive periods in non-pregnant women with past menses [54], which can be caused by polycystic ovary syndrome^a^, hypothalamic amenorrhea^b^, hyperprolactinemia, or primary ovarian insufficiency [55]			
Define *naturally menstruating* women as those who experience menstruation, with menstrual cycle lengths ≥ 21 days and ≤ 35 days, but without confirmed ovulation [ovulation was not confirmed by urinary LH surge or verified by hormone concentrations via blood sample analysis]	Increase accuracy and validity of the population definition	Reduce unfounded assumption that ovulation, and thus eumenorrhea, has been established Reduce between study variability describing the population studied	Increase timescale of the study in order to recruit participants who fit the criteria
A priori exclusion of participants with self-reported or diagnosed menstrual irregularities for eumenorrheic studies. Menstrual irregularities refer to perturbations of the eumenorrheic menstrual cycle, such as amenorrhea, anovulation, oligomenorrhea etc	Increase homogeneity of hormonal profiles	Reduce between participant variability in hormone status Increase validity of the data	Reduce availability of eligible participants for eumenorrheic studies
A posteriori exclusion of participants with observed or implied menstrual irregularities for eumenorrheic studies			Increase number of participants who need to be excluded (during or retrospectively)
No HC use ≥ 3 months prior to recruitment for study on eumenorrheic participants	Increase likelihood that an eumenorrheic cycle and its typical hormonal profile has been re-established	Reduce occurrence of atypical hormonal profiles not fitting the eumenorrheic definition	Reduce availability of eligible participants for eumenorrheic studies Increase timescale of the study as the condition needs to be met prior to recruitment
HC use ≥ 3 months prior to recruitment for HC studies	Increase likelihood that eumenorrheic cycle has been removed and replaced by a hormonal profile indicative of HC use	Reduce occurrence of atypical hormonal profiles not fitting the characterisation of HC users	
Define *HC users* as those taking any type of contraceptive capable of altering the endogenous hormonal milieu. Please note that HC also influence other aspects of metabolism, which are beyond the scope of this paper	Increase accuracy and validity of the population definition	Reduce between study variability in describing the population studied	N/A
Report the type (e.g., OCPs, implants, injections, intrauterine devices/coils that are hormone releasing and NOT copper-based, vaginal rings, contraceptive transdermal patches) and formulation (e.g., mono, bi or triphasic; combined or progesterone-only; names and concentration of exogenous hormones) of HC used	Increase reliability of studies Increase validity of findings	Reduce between participant variability in hormone status Reduce between study variability in describing the population studied	Increase number of participants who need to be excluded as they do not know the exact type or formulation of HC used
One brand/type of OCP per group of participants [56]. Clearly identify exogenous hormone names and concentrations in each OCP, as well as androgenicity	Increase homogeneity of hormonal profiles (both endogenous and exogenous)		Increase timescale of the study whilst trying to recruit a sufficient sample size on the same brand/type of OCP Reduce generalisability of the findings
Define the *first trimester* as first 13 weeks of pregnancy [54]	Increase accuracy and validity of the population definition		Increase timescale of the study in order to recruit participants who fit the criteria
Define the *second trimester* as the time from week 14 to week 27 of pregnancy [54]		Reduce between participant variability in hormone status Reduce between study variability in describing the population studied	Increase timescale of the study in order to recruit participants who fit the criteria
Define the *third trimester* as the time from 27 weeks of pregnancy onwards [54]			
Define *full term* as when a pregnancy is a normal duration (i.e., 37–42 weeks gestation) [54]			
Define postpartum as the 12 months following parturition			
State gestation (i.e., the length of time, in days or weeks, that a baby is in the uterus)		Reduce between study variability in describing the population studied	Increase number of participants who need to be excluded as they do not know their exact gestational stage
State gravidity (i.e., number of times that a woman has been pregnant, including miscarriages and abortions)			Increase timescale of the study in order to recruit women willing to state this number (can be a sensitive issue)
State parity (i.e., number of times that a woman has given birth to a foetus with a gestational age of 24 weeks or more, regardless of whether the child was born alive or was stillborn)			
State singleton or multiple pregnancy			
Define *peri-menopause* as the time around the occurrence of the menopause when the ovaries gradually produce less oestrogen [57]	Increase accuracy and validity of the population definition	Reduce between study variability in describing the population studied	Need to be aware of other physical indicators of menopause (e.g.*,* hot flushes, vaginal dryness, emotional changes)
Define *menopause* as the time when menstruation surceases; i.e., characterised by sporadic amenorrhea. *Please note that this is the transitional time between peri- and post-menopause			Increase timescale of the study in order to recruit participants who fit the criteria
Define *post-menopause* as the time after which a woman has experienced 12 consecutive months of amenorrhea and is characterised by < 118 pmol∙L^−1^ of oestrogen, < 4.4 nmol∙L^−1^ of progesterone and follicle stimulating hormone > 25 IU∙L^−1^ [57]	Increase homogeneity of hormonal profiles Increase consistency of terminology	Reduce between participant variability in hormone status Reduce between study variability in describing the population studied	Reduce availability of eligible participants Increase timescale of the study if the condition needs to be confirmed prior to the commencement of data collection Increase number of participants who need to be excluded (during or retrospectively) if the condition was not confirmed prior to commencement of the study
Consider menopausal symptoms [58]	To limit the effects of menopausal symptoms on outcomes	Reduce the likelihood that effects are indirectly due to symptoms rather than directly due to changes in hormones	Increase the time burden to identify participants with no, or a consistent set of, menopausal symptoms capable of affecting the intended outcome
Define *HRT users* as those taking any type of HRT capable of altering the endogenous hormonal milieu (e.g., tablets, skin patches, gels, implants, vaginal creams, pessaries or rings; combined or oestrogen only; cyclical or continuous)	Increase accuracy and validity of the population definition	Reduce between study variability in describing the population studied	N/A
Report the type and formulation of HRT used	Increase reliability of studies Increase validity of findings	Reduce between participant variability in hormone status Reduce between study variability in describing the population studied	Increase number of participants who need to be excluded as they do not know the exact type or formulation of HRT used

Considerations without a reference have been developed by the authors for this statement

*HC* hormonal contraceptives, *HRT* hormone replacement therapy, *OCP* oral contraceptive pill, *LH* luteinising hormone, *NA* not applicable

^a^Please note that polycystic ovary syndrome affects between 2.2% and 26% of women worldwide, based on data from the World Health Organisation [59]

^b^Please note that for women with functional hypothalamic amenorrhea (especially with the female athlete triad), this condition should be immediately corrected

### Experimental Design

The experimental design employed in studies with women as participants is often poorly considered, described, controlled and/or executed, which is mainly due to a lack of understanding of the hormonal changes experienced across the lifespan in women. In addition, studies involving women as participants usually require repeated measures due to the acute (e.g., ultradian, circadian and infradian rhythms) and chronic fluctuations (e.g., pregnancy, menopause, HC and HRT usage) in reproductive hormones over time, unlike in studies with men as participants who have relatively stable (i.e., outside of circadian variation) sex hormone profiles following puberty. Table 2 outlines some methodological approaches that could be employed at various stages of the life cycle (i.e., puberty, menstrual cycle and irregularities, hormonal contraception, pregnancy and menopause) in women.

**Table 2:** Summary table of the considerations related to experimental design for women’s studies from puberty to post-menopause

Consideration	Rationale (intended to…)	Pros (could…)	Cons (could…)
Take into account the changes in androstenedione prior to the onset of puberty (e.g., the initial increase in androstenedione has been noted 18 to 12 months prior to the onset of puberty) [60]	Increase breadth of data on reproductive ageing by considering the peri-pubertal period	Increase understanding of the peri-pubertal changes in physiological functioning and athletic performance	Increase timescale of the study in order to identify and group participants along these spectrums
Take into account the changes in oestrogen prior to the onset of puberty (e.g., the initial increase in oestrogen has been noted 12 and 6 months prior to the onset of puberty) [60]			
Take into account the time scale of establishing a eumenorrheic cycle: menarche follows an anovulatory cycle; menstrual cycles during the 1st year after menarche are typically irregular and anovulatory, ranging in duration from 21 to 45 days; by 3 years post-menarche, > 90% of girls have ≥ 10 menstrual cycles per year with an average menstrual interval of 36.5 days; cycles can remain irregular until the 5th year post-menarche [61]	Reduce assumption that once menarche has been initiated all girls have fully eumenorrheic cycles	Reduce between participant variability in hormone status	
Define menstrual cycle phases based on hormonal profiles (see Table 3), verified by blood analysis with inter and intra-assay variability reported or other robust biochemical methods [62]	Increase reliability of studies Increase validity of findings	Reduce likelihood of grouping non-homogenous hormonal profiles Reduce inconsistency in phase definitions between studies	Increase timescale of the study in order to recruit participants who are willing to undertake blood sampling Increase cost of the study
Track and establish menstrual cycle characteristics for ≥ 2 months prior to testing. Tracking can be achieved by denoting the first and the last day of menstruation on a calendar for each cycle. Corroboration can be achieved by confirmation of ovulation and hormone concentrations		Reduce within participant variation in menstrual cycle characteristics Reduce likelihood of including participants with menstrual irregularities in eumenorrheic studies Increase ability to accurately predict testing timepoints (i.e., phases)	Increase timescale of the study due to the long lead-in time Increase burden on participants to track their cycles before the experimental aspect of the study
Outcome measures should be repeated in a second cycle		Reduce variability of the data	Increase timescale of the study due to the repeated measures Increase burden on participants to repeat all of the testing sessions
Use urinary ovulation kits to establish the mid-cycle surge in LH; visual confirmation should be provided to the researcher [58, 63, 64]		Reduce risk of including anovulatory women in eumenorrheic studies Reduce chance of a false positive result by the participant from an at-home interpretation	Increase chance of missing a positive ovulation result in participants who do not comply or adhere to conducting the test at the same time of day Increase likelihood of overlooking LPD as this method does not exclude LPD cycles
Apply a posteriori exclusion of data from testing timepoints which do not comply with the theoretical (see Fig. 2 and Fig. 3) or stipulated (see Table 3) differences in reproductive hormone concentrations in menstrual cycle studies	Ensure that the intended reproductive profiles were assessed	Reduce likelihood of grouping non-homogenous hormonal profiles	Increase number of participants who need to be excluded (retrospectively) as a result of not fitting the inclusion criteria
Stipulate and take into account OCP-taking (i.e., active OCP) days and OCP-free (i.e., inactive/placebo OCP) days: (i) The endogenous concentration of oestrogen and progesterone rises during the OCP free/inactive/placebo days [65, 66] (ii) The concentration of exogenous hormones increases during active OCP intake: for example, for a combined monophasic OCP ethinyl estradiol (a type of exogenous oestrogen) increases twofold from day 1 of active OCP to day 21 [66, 67] and progestin increases threefold from day 1 of active OCP to day 8–11 and then maintains that level [67, 68]	Increase homogeneity of hormonal profiles	Reduce between participant variability in hormone status	Increase timescale of the study if several conditions need to be assessed
Take into account the rising concentrations of oestrogen and progesterone throughout each trimester of pregnancy			Increase timescale of the study in order to identify and group participants along this spectrum
Take into account the large variation in hormonal profiles associated with the peri-menopause, menopause and post-menopause, thus treating these as separate categories of women based on the criteria outlined in Table 1			

Considerations without a reference have been developed by the authors for this paper. *OCP* oral contraceptive pill,* LPD* luteal phase deficiency, *LH* luteinising hormone

**Table 3:** Proposed menstrual cycle phase definitions based on hormonal profiles (see Fig. 4)

Recommendation	Rationale (intended to…)	Pro	Con
**Phase 1:** indicated by the onset of bleeding until day 5 Oestrogen and progesterone levels are low	Capture the lowest concentrations of oestrogen and progesterone	Easy to determine due to obvious physical cue (i.e., bloody discharge)	Can be difficult to predict in those with variable cycle length therefore requiring reactive testing sessions (i.e., participant alerting the researcher on day 1 of bleeding and then both parties having availability for testing within the next 4 days)
**Phase 2:** occurs in the 14–26 h prior to ovulation and the LH surge Oestrogen higher than during phase 1, 3 and 4 and progesterone higher than during phase 1, but lower than 6.36 nmol·L^−1^	Capture the highest oestrogen concentration, while progesterone remains low	Enables the biggest difference between oestrogen and progesterone to be investigated	Difficult to predict without daily blood samples for the determination of oestrogen and progesterone
**Phase 3:** indicated by a positive urinary ovulation kit and lasts 24–36 h Oestrogen higher than phase 1 but lower than phase 2 and 4 and progesterone higher than phase 1 but lower than 6.4 nmol·L^−1^	Capture a medium oestrogen concentration, while progesterone remains low	Easy to establish due to the positive LH surge captured by the ovulation kit	Relies on having multiple ovulation kits available for each participant (cost) and requires reactive testing sessions (i.e., participant alerting the researcher to the positive result and then both parties having availability for testing within the next 24–36 h)
**Phase 4**: + 7 days after ovulation has been confirmed Oestrogen higher than phase 1 and 3 but lower than phase 2 and progesterone > 16 nmol·L^−1^	Capture the highest concentration of progesterone and a high concentration of oestrogen	Easy to establish in those with eumenorrheic cycles as it typically occurs within 7 days of confirmed ovulation	Relies on the confirmation of ovulation

*LH* luteinising hormone

These recommendations have been developed using information from the following sources: McGovern et al. [69]; Tsampoukos et al. [70]; Janse de Jonge et al. [48]; Elliott-Sale et al. [71]. The phases described in Table 3 are referred to as number (i.e., 1–4), rather than names (i.e., follicular, ovulatory and luteal), to reduce the misidentification or mislabelling of phases. This should ensure that phases are described based on quantifiable metrics rather than ambiguous terms. In order to draw comparisons with previous literature the following matches can be used: phase 1 with the ‘early follicular’ phase; phase 2 with the ‘late follicular’ phase; phase 3 with the ‘ovulatory’ phase; and phase 4 with the ‘mid-luteal’ phase. To date, no consensus has been reached within sport and exercise science on the nomenclature used to describe menstrual cycle phases and their corresponding hormonal profiles

**Fig. 2:**
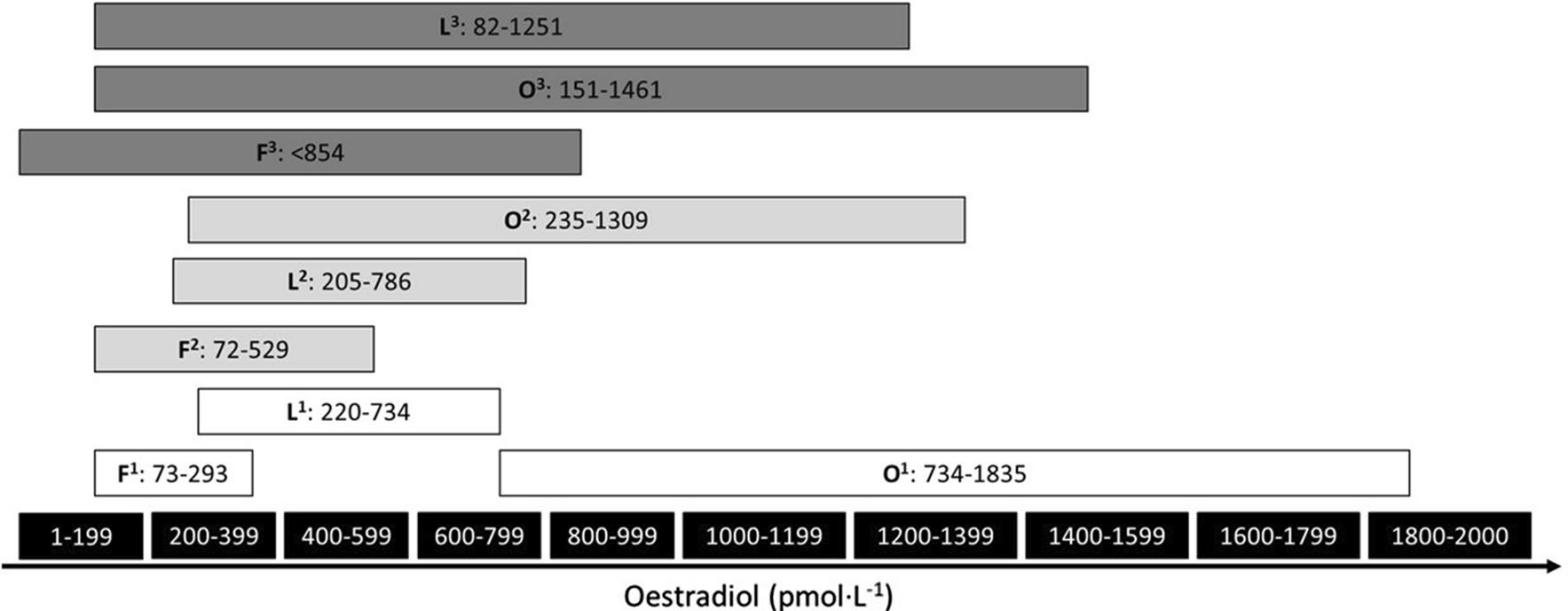
Oestrogen reference ranges from three different sources^1,2,3^ for the follicular (F), ovulatory (O) and luteal (L) phases of the menstrual cycle. All sources are clinical reference ranges from different National Health Service Trusts in the UK and are based on the manufacturers' laboratory reference ranges. This figure illustrates the differences in the reference ranges for each of the three prevailing oestrogen phases of the eumenorrheic menstrual cycle and suggests that additional context is required (e.g., blood sample taken on day two of bleeding, thus allowing the comparison with the follicular range; blood sample taken 12 h after ovulation was denoted by a positive urinary luteinising hormone test kit, thus allowing comparison with the ovulatory range; blood sample taken seven days after ovulation was denoted by a positive urinary luteinising hormone test kit, thus allowing comparison with the luteal range values)

**Fig. 3:**
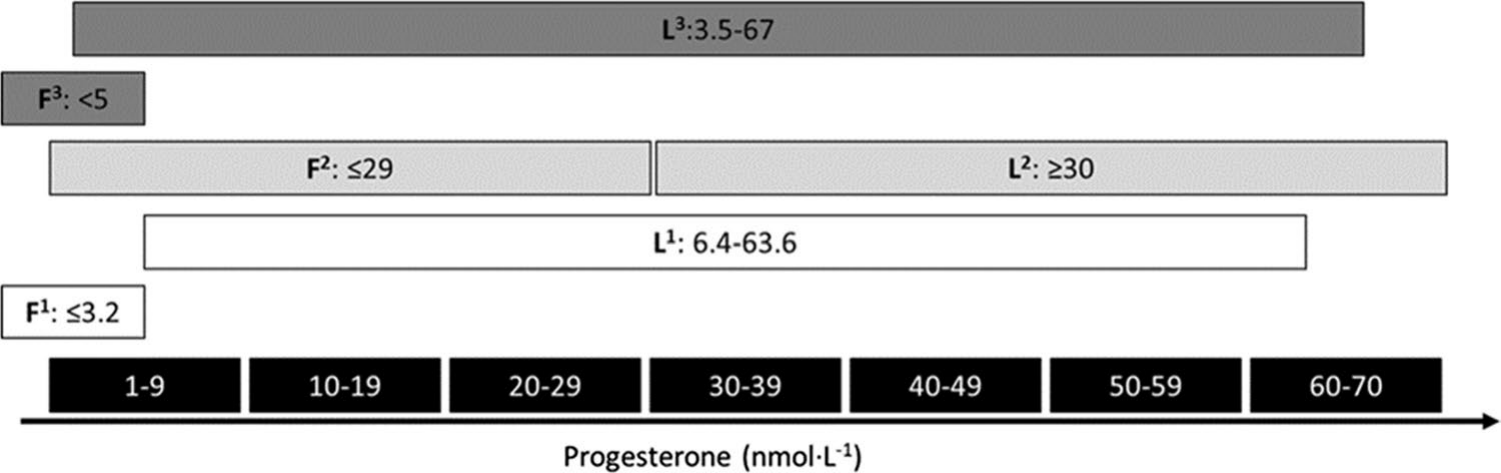
Progesterone reference ranges from three different sources^1,2,3^ for the follicular (F) and luteal (L) phases of the menstrual cycle. All sources are clinical reference ranges from different National Health Service Trusts in the UK and are based on the manufacturers' laboratory reference ranges. This figure illustrates the differences in the reference ranges for each of the two prevailing progesterone phases of the eumenorrheic menstrual cycle and suggests that additional context is required (e.g., blood sample taken on day two of bleeding, thus allowing the comparison with the follicular range; blood sample taken seven days after ovulation was denoted by a positive urinary luteinising hormone test kit, thus allowing comparison with the luteal range values)

**Fig. 4:**
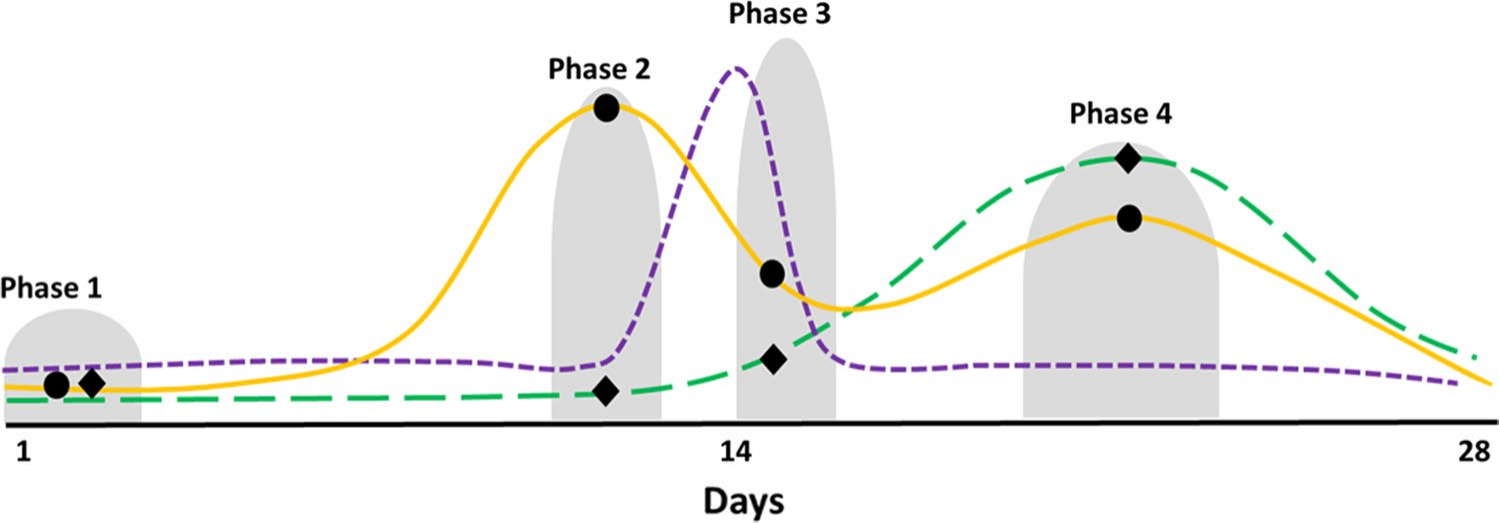
Visual overlay of the hormonal changes across an idealised 28-day menstrual cycle indicating when each phase begins and ends as described in Table 3. The solid gold line represents oestrogen the short dash purple line represents luteinising hormone and the long dash green line represents progesterone. The black dots represent the mean concentration of oestrogen during each phase and the black diamonds represent the mean concentration of progesterone in each phase

One particular aspect of research design that has continued to cause debate amongst authors and disparity between studies is the nomenclature associated with the phases of the menstrual cycle and menopausal status [57]. In the simplest terms, the menstrual cycle can be divided into two phases that are separated by ovulation; follicular (begins at the onset of menses) and luteal (post ovulation). These phases can be further divided into the early, mid and late phases for each, with or without the inclusion of ovulation as a discrete phase. As such, studies involving eumenorrheic women report phases on a continuum from two to seven. Furthermore, the basis for this taxonomy is often unclear; i.e., the phases are linked to poorly defined or undefined ovarian steroid profiles. Rather than continue to dispute the name of menstrual cycle phases, a consensus is needed on the hormonal profiles of interest, such that testing timepoints and the determination of eligible data can be based on an a priori decision. Table 3 and Fig. 4 show four distinct hormonal profiles, which represent significant changes in both oestrogen and progesterone concentrations. These four profiles could be used to investigate the effects of ovarian steroids on physiology, health and performance.

### General Guidance

Some methodological issues can affect all sport and exercise science studies with women as participants; these points are described in Table 4. One consideration is the use of “unclassified” women, viz. where the reproductive profile of the participants is not stated. In some studies, it is relevant *to describe* the menstrual characteristics of the participants, but not necessary *to measure* ovarian steroid levels. It is important to balance the internal and external validity of a study, such that the translational reach and impact of the data are not limited. The research design should be suitable to answer the research question. For example, if a study is designed to examine the effects of menstrual cycle phase on an outcome, then biochemical confirmation of the phase is warranted alongside self-reported menstrual characteristics (e.g., last menses), whereas if a study is designed to examine the physical/performance characteristics of a female sports team, then a description of the population (i.e.*,* solely self-reported menstrual cycle phase) may be sufficient. As such, the research question should clearly indicate if the intention is to attribute an outcome to a specific hormonal profile or to observe the characteristics/responses of a clearly defined, mixed group of sportswomen or exercisers.

**Table 4:** General research considerations

Consideration	Rationale (intended to…)	Pros (could…)	Cons (could…)
Employ a single-blind design; although participants cannot be blinded, the researcher can be blind to the intended testing timepoint	Protect against bias	Reduce unintentional bias from the researcher to the participant	Increase staffing as an independent person is needed to undertake the blinding process
Do not use unclassified (i.e., not stating reproductive status) women as participants even if you are not concerned by the potential influence of reproductive hormones on your outcome measure	Increase homogeneity of hormonal profiles	Reduce between participant variability in hormone status Increase validity of the data	Reduce the availability of eligible participants
Standardise, by quantitative means, time of day, prior exercise, caffeine ingestion, dietary intake and nutritional supplementation, alcohol consumption and smoking as these have been shown to affect the concentration of reproductive hormones	Control extraneous variables	Allow the investigation of X on Y, without the influence of Z	Adds more requirements on the participants by asking them to standardise a large number of variables over a set period of time
Use the term “withdrawal bleed” rather than “period” when referring to the bleed experienced by OCP users	Stop OCP users misidentifying themselves as eumenorrheic based on bleeding patterns	Education; informing women and researchers about the differences between hormonal contraceptive users and non-users	
Do not use the terms “menstrual cycle” and “periods” synonymously	Dispel the myth that they are the same things and that these terms can be used interchangeably	Education; informing women and researchers about menstrual cycles and how periods are just one aspect of that cycle	
Do not impose any menstrual cycle language upon HC users (*i.e*., trying to match certain days of the OCP cycle with eumenorrheic phase descriptions, e.g., calling the first five days of OCP taking as the early follicular phase of the menstrual cycle)	Prevent confusion between hormonal contraceptive users and non-users	Allows HC users to describe their own status without adding unnecessary complexity or without misperception	
Report gestational age based on ultrasound dating rather than on last menstrual period	Increase the accuracy of reporting of gestational age	Reduce the ambiguity in defining participants in studies involving pregnant women	
Consider the timescale for resumption of eumenorrheic cycles following childbirth (i.e., in the postpartum period), given that this varies considerably between women	Reduce the assumption that all postpartum women who do not use hormonal contraceptives have eumenorrheic cycles	Reduce ambiguity in defining participants in studies involving postpartum women	
Do not use the term post-menopausal based on participants’ age solely	Protect against including irrelevant participants and to increase the homogeneity of hormonal profiles	Reduce between participant variability in hormone status Increase validity of the data	Increase the time and economic burden to correctly identify and confirm post-menopausal status
Do not report gynaecological age (i.e., number of years from menarche to recruitment in the study) as a characteristic of menstrual function (i.e., to illustrate the number of years with eumenorrheic menstrual cycles)	Reduce the assumption that the time between menarche and recruitment is filled with eumenorrheic cycles	Reduce ambiguity in defining participants in studies involving eumenorrheic women	
Include an online supplement with additional in-depth information about reproductive status; e.g., data from questionnaires on menstrual cycle status or hormonal contraceptive use, blood marker data, etc	Provide data that can be used for future meta-analyses in studies with women as participants	Quickly increase our understanding of female physiology in relation to sport and exercise science	

The considerations in this table have been developed by the authors for this paper

*HC* Hormonal contraceptives, *OCP* oral contraceptive pill OCP

## Discussion

### Definition of ‘Woman’

Herein cisgender females are described and defined based on ovarian steroid concentrations. Transgender females and women with differences in sexual development have not been characterised but require and deserve further clear research parameters to better understand their physiology. The diversity in female reproductive endocrinology makes it difficult to title and define women based on reproductive hormonal profile alone. As such, it can be challenging for researchers to decide on the inclusion/exclusion criteria for participant recruitment; determine the methods by which this criteria can be established; and use the eligibility criteria to inform the research design.

- Example 1; a researcher wishing to investigate potential effects of the menstrual cycle on exercise performance needs to decide: if they will use eumenorrheic or naturally menstruating women (see Table 1 for delineation of terminology); how they will confirm eumenorrhea; when in the cycle they will test to ensure different hormonal environments.
- Example 2; a researcher wishing to use oral contraceptive pill (OCP) users will need to decide which role they will fulfil (i.e.*,* control or experimental sample [56]):
- OCP users can be used as a control group, wherein cyclical fluctuations in oestrogen and progesterone experienced during the menstrual cycle can be compared to consistently downregulated sex hormone levels. The researcher then needs to decide whether to compare with the active pill phase or the non-active pill phase. For example, if the non-active pill phase is compared with the early follicular phase, then it is likely that the hormonal environments will be very similar between the two groups. If the mid-luteal phase is compared with the active pill phase, the hormonal environments between groups will be different. The researcher will also need to consider the type of OCP being used (i.e., oestrogen and progestin formulations and concentrations; monophasic, biphasic, triphasic) and the duration of usage, ensuring this is similar for the whole control group of OCP users.
- OCP users can also be considered as an experimental group, wherein (i) the effects of low endogenous oestrogen and progesterone levels can be investigated and/or (ii) the effects of exogenous oestrogen and progestin can be examined. In this case the researcher needs to decide which hormonal environments to compare, early in the non-active pill phase (with low endogenous and exogenous hormone levels), late in the non-active pill phase (with increasing endogenous oestrogen and low exogenous hormones), early in the active pill phase (with increasing exogenous hormone levels) or late in the active pill phase (with highest exogenous hormone levels). Again, the researcher will need to consider the type of OCP being used and ensure this is similar for the whole control group of OCP users and/or consider comparison between different types of OCP.

Example 3; a researcher wishing to conduct menopause-related study needs to decide which menopausal phases to include and how to define those phases; whether or not to include HRT users or those with ovarian concerns that have resulted in hormonal conditions similar to menopause; if post-menopausal women are included, how to define the time that the participants have been post-menopausal [72].

As such, we propose that:

(i) sample populations are chosen based on the research question;
(ii) participants are clearly named and described based on their reproductive characteristics;
(iii) hormonal profiles are used to inform the experimental design.

These steps should help alleviate the ambiguity surrounding the classification of women previously seen in sport and exercise studies that has led to considerable sampling heterogeneity and has hindered meaningful comparison between studies.

### Study Validity

The validity of studies, or lack thereof, in sport and exercise science using women as participants is rarely discussed. The considerations described herein are designed to increase the internal validity through adequate quality control as a result of stringent recruitment strategies (e.g., using predetermined population definitions and strict inclusion/exclusion criteria) and appropriate experimental design (e.g., a priori classification of hormonal profile associated with each testing timepoint and *post priori* exclusion of data not meeting the stipulated terms). These measures should help to ensure that the results reflect the truth in the population studied rather than methodological errors [73]. At present, given the lack of clarity on either the direction or magnitude of potential effects of ovarian steroids on physical performance, maximising internal validity should be considered a research priority. The external validity of studies involving women as participants is often more difficult to increase due to the large inter and intra-individual variability in ovarian hormone concentrations; by reducing some of this variation through precise recruitment and testing, the external validity is lessened, and thus generalisability is reduced. These issues can be somewhat overcome through field-based testing and by testing entire teams, which could help maximise the translational potential of performance-based research in women.

### Inclusion of Research with Women as Participants

Until recently, women were less represented in elite sport. Women accounted for only 2.2% of the athletes in the Paris Olympics in 1900, but they are predicted to account for an estimated high of 48.8% in the next Olympic Games [74]. Therefore, the time is right to shift more attention to women as participants in sport and exercise science research, especially given that often the research findings that are based on male participants are neither applicable nor generalizable to women. It is not appropriate to exclude women from sport and exercise science research for any reason, especially on the basis of convenience, i.e. excluding women as participants due to the potential confounding effect of female hormone fluctuations, or cost, i.e., excluding women as participants because they can be more expensive than men to study due to the extra time (e.g., repeated measures across a menstrual cycle) and resources (e.g., blood samples for the determination of ovarian steroid levels) needed to produce high-quality data. Unfortunately, it is evident that many researchers have avoided using female participants with data indicating just 4 to 13% of articles from leading journals in sports and exercise science included only women as participants [15]. So even though some research has focussed on the effects of ovarian steroids on performance, health and physiology, the small quantity of research in this area limits our understanding of the topic. Furthermore, the heterogeneity in study populations (e.g.*,* eumenorrheic, OCP user, post-menopausal) and large variation in research designs (e.g.*,* definition of menstrual cycle phase, oral contraceptive pill consumption or withdrawal) has resulted in many conflicting findings in sport and exercise science research with women as participants. To improve our understanding of many female specific topics, undoubtedly more high-quality research is warranted to overcome the heterogeneity and ambiguity seen in the limited number of studies in this area to date. Women account for half the world-wide population, therefore a willingness to study women as participants in sport and exercise science needs to be fostered and funding needs to be made available to achieve equivalence in knowledge about the female sex.

### Limitations of the Statement

Although this paper has covered many aspects of the reproductive ageing female (i.e., from puberty to the menopause), it has not covered every perturbation of the menstrual cycle [75] or every type of hormonal contraceptive [76] or all aspects of (post) menopausal hormonal therapy. In addition, medications known to influence the hypothalamic-pituitary-ovarian axis are beyond the scope of this statement [77].

## Conclusions

In order to improve the quality of future research in sport and exercise science with women as participants, more researchers need to accept the challenges of working in this area and embrace the means and mechanisms to produce high quality scientific studies on essentially half the world’s population. The considerations presented in this paper are not intended to be the panacea for studies on sportswomen; instead they are intended to guide readers to adopt good practice in this area and prompt further consideration of the issues involved in women-based sport and exercise science studies. As every woman’s menstrual cycle is different and can change across the lifespan, there might never be a universal blueprint that research studies and practitioners can exclusively use to direct training and performance; however, if we understand and appreciate how ovarian hormones affect different physiological systems, we can use this knowledge to tailor exercise programmes for women in response to the reproductive events throughout their lifespan.
